# Triboelectric Nanogenerators and Hybridized Systems for Enabling Next-Generation IoT Applications

**DOI:** 10.34133/2021/6849171

**Published:** 2021-02-26

**Authors:** Qiongfeng Shi, Zhongda Sun, Zixuan Zhang, Chengkuo Lee

**Affiliations:** ^1^Department of Electrical & Computer Engineering, National University of Singapore, 4 Engineering Drive 3, Singapore, Singapore 117583; ^2^Center for Intelligent Sensors and MEMS, National University of Singapore, 4 Engineering Drive 3, Singapore, Singapore 117583; ^3^Smart Systems Institute, National University of Singapore, 3 Research Link, Singapore, Singapore 117602; ^4^NUS Graduate School for Integrative Science and Engineering, National University of Singapore, Singapore, Singapore 117456

## Abstract

In the past few years, triboelectric nanogenerator-based (TENG-based) hybrid generators and systems have experienced a widespread and flourishing development, ranging among almost every aspect of our lives, e.g., from industry to consumer, outdoor to indoor, and wearable to implantable applications. Although TENG technology has been extensively investigated for mechanical energy harvesting, most developed TENGs still have limitations of small output current, unstable power generation, and low energy utilization rate of multisource energies. To harvest the ubiquitous/coexisted energy forms including mechanical, thermal, and solar energy simultaneously, a promising direction is to integrate TENG with other transducing mechanisms, e.g., electromagnetic generator, piezoelectric nanogenerator, pyroelectric nanogenerator, thermoelectric generator, and solar cell, forming the hybrid generator for synergetic single-source and multisource energy harvesting. The resultant TENG-based hybrid generators utilizing integrated transducing mechanisms are able to compensate for the shortcomings of each mechanism and overcome the above limitations, toward achieving a maximum, reliable, and stable output generation. Hence, in this review, we systematically introduce the key technologies of the TENG-based hybrid generators and hybridized systems, in the aspects of operation principles, structure designs, optimization strategies, power management, and system integration. The recent progress of TENG-based hybrid generators and hybridized systems for the outdoor, indoor, wearable, and implantable applications is also provided. Lastly, we discuss our perspectives on the future development trend of hybrid generators and hybridized systems in environmental monitoring, human activity sensation, human-machine interaction, smart home, healthcare, wearables, implants, robotics, Internet of things (IoT), and many other fields.

## 1. Introduction

With the gradual rollout of the 5G (5th-generation mobile networks) technology across the world, the role of the Internet of things (IoT) is becoming more and more essential in both industrial and commercial developments [1]. Entering the IoT era, wireless and portable electronics are undergoing explosive advancement, with the total number increasing tremendously and power consumption decreasing significantly. Affected by the nature of incredibly huge numbers, small power consumption, and ultrawidely distributed location of IoT devices, energy supply in the new era should be changed from the centralized, immobile, ordered, and large-scale mode to the distributed, mobile, in situ, and small-scale mode. Yet batteries as the primary choice of conventional energy supply exhibit apparent drawbacks such as the limited lifespan that needs frequent replacement or recharging, large volume and weight, rigidity, biological incompatibility, and environmental pollution. Thus, to meet the above requirements, the ideal energy supply units should possess the characteristics of portability, sustainability, miniaturization, wearability, and implantability depending on the applications [2–7]. In this regard, scavenging energy from the ambient surroundings by energy harvesters can provide a green, portable, and sustainable solution.

There exist various types of energy forms that are normally wasted in the environment, including mechanical energy associated with diverse nature vibrations and human activities, thermal energy, and solar energy. Accordingly, different types of energy transducing mechanisms and generators (i.e., energy harvesters) have been developed to scavenge these wasted energies, such as the electromagnetic generator (EMG), piezoelectric nanogenerator (PENG), and triboelectric nanogenerator (TENG) for mechanical energy, the thermoelectric generator (TEG) and pyroelectric nanogenerator (PyENG) for thermal energy, and the solar cell (SC) for solar/light energy. In most scenarios, multitype energy forms coexist in the ambient environment, and the strength of a single-type energy form may vary significantly from time to time, such as solar energy varying with time and weather, random wind/wave energy, and different human activities. Therefore, generators employing a single-energy transducing mechanism may greatly suffer from the unstable energy source, low energy utilization rate, low conversion efficiency, and low adaptability in different scenarios. On the other hand, hybrid generators utilizing integrated transducing mechanisms can be more effective in harvesting both single-type and multitype energies. Through synergetic designs, hybrid generators can compensate for the shortcomings of each mechanism, improve the space utilization efficiency, enhance the energy utilization rate, and thus can be applied in diverse scenarios as the energy supply units [8–10].

Among different energy forms, mechanical energy is one of the most ubiquitous energy sources, widely existing in water wave, wind, sound/ultrasound, machinery vibration, human motions, etc. Considering that most IoT devices are adopted in the environment- or human-relative applications where they exhibit abundant mechanical energy, generators with mechanical energy harvesting ability will be most desirable. Since the first invention in 2012 by Prof. Wang and his team [11], the TENG technology has been extensively explored for mechanical energy harvesting, due to its superior advantages of high output performance, versatile operation modes, broad material availability, wearable/implantable compatibility, simple fabrication, high scalability, and low cost [12–15]. Benefitting from these merits, integrating TENG with other transducing mechanisms yields a promising research direction for developing hybrid generators, which has received flourishing development in the past few years [16–19]. Furthermore, after integrating the TENG-based hybrid generators with power management circuitry, energy storage units, and functional components, a variety of hybridized systems with self-sustainability can be achieved for broad applications.

Here, in this review, we systematically introduce the key technologies and the recent progress in the TENG-based hybrid generators and hybridized systems: (1) principles of different types of transducing mechanisms and generators; (2) strategies for enhancing the output performance of TENGs; (3) applications of TENG-based hybrid generators in the outdoor, indoor, and on-human-body scenarios; (4) strategies for achieving efficient power management and energy storage; and (5) demonstrations of functional and self-sustainable hybridized systems for various applications. These sections summarize the development trend of the TENG-based hybrid generators and hybridized systems in the aspects of operation principles, structure designs, optimization strategies, power management, system integration, and applications. In the end, conclusions and perspectives on the existing challenges and future trends are also provided, which give a glimpse of the further development of hybrid generators and hybridized systems in the IoT era.

## 2. Transducing Mechanisms and Generator Principles

**Figure 1:**
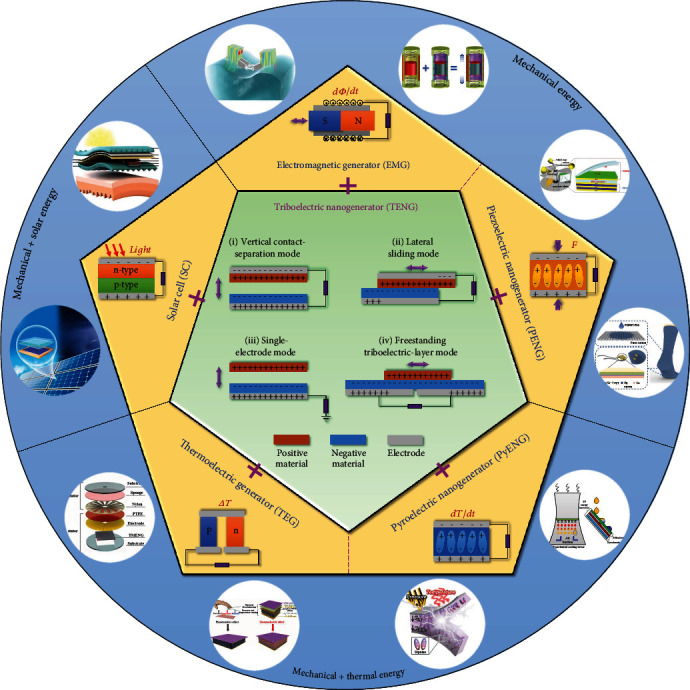
Overview of different transducing mechanisms for converting different forms of energies into electricity and their respective generators for hybrid generator integration. Reproduced with permission from Refs. [65, 73, 107, 126, 132–135, 172, 177], copyright 2019 Wiley-VCH, 2020 Elsevier B.V., 2018 Elsevier B.V., 2019 American Chemical Society, 2020 Elsevier B.V., 2020 Elsevier B.V., 2020 American Association for the Advancement of Science, 2018 Wiley-VCH, 2020 American Chemical Society, and 2019 Elsevier B.V.

To scavenge different types of ambient available energies, generators based on different transducing mechanisms have been developed. As shown in Figure 1, TENG/EMG/PENG, PyENG/TEG, and SC are the most common generators employed for mechanical, thermal, and solar/light energy harvesting, respectively. Through synergetic integration, their hybrid generators can be further employed for complementary and effective energy harvesting of both single-type and multitype energies.

### 2.1. TENG

The first TENG was invented in 2012, based on the coupling effect of contact electrification (triboelectrification) and electrostatic induction, which can convert mechanical energy into electricity [11]. The origin of TENG and other types of nanogenerators (e.g., PENG and PyENG) is Maxwell's displacement current theory, i.e., external current induced by a time-varying electric field in the nanogenerators [20]. More specifically, when two dissimilar materials contact with each other, due to their different electron affinity, surface charges are generated at the contact interface. Then, upon separation, the built-up electric potential will drive electrons on the respective electrodes to flow in the external circuit until a new balance is achieved. If the two materials are brought into contact again, the electric potential will disappear and electrons will flow in a reverse direction. Thus, under periodic contact and separation, alternating current (AC) can be generated on the external load, and a rectification circuit is normally required for energy storage to convert the AC output into the direct current (DC) output [21–23]. There are also some advanced designs of DC-TENGs that do not need a rectification circuit before energy storage [24–28], but they exhibit more complicated structures and most developed TENGs are still AC-based. According to a material's ability to lose or accept electrons (electron affinity), different materials can be arranged into a sequence called the triboelectric series, from the most positive material to the most negative material [29, 30].

Along these years, different models including the electron-cloud-potential-well model have been developed to explain the origin of contact electrification among polymers, metals, semiconductors, and even liquids [31–34]. It is revealed that in most cases, electron transfer is the dominating effect of the contact electrification process. Specifically, for the solid-liquid contact electrification, a two-step formation of the electric double layer (EDL) at the solid-liquid interface is proposed by Prof. Wang, which is also known as the Wang model [34, 35]. The Wang model indicates that the electron transfer is required at the very first contact to create the first layer of electrostatic charges on the solid surface, and then, the ion transfer in solution dominates in the second step due to the electrostatic interactions with the charged solid surface. The surface charge density, as one of the most important parameters determining the output performance of TENGs, can be enhanced through several strategies, e.g., proper material selection (contact materials with a larger difference in electron affinity), surface modification (like micro-/nanostructures, chemical treatment, and ion injection), structural optimization, middle layer insertion, and circuity assistance [36–38]. With the generated surface charges, the output voltage on an external load can be given by [39]

(1) $$\begin{array}{c} V_{\text{load}}=V_{\text{oc}}-\frac{Q}{C\left(x\right)}, \end{array}$$

where *V*_oc_ is the open-circuit voltage that is greatly determined by the surface charge density, *Q* is the transferred charges across the two electrodes, and *C*(*x*) is the instant capacitance of the TENG at a separation distance of *x*. In general, there are four basic operation modes of TENG, i.e., vertical contact-separation mode, lateral sliding mode, single-electrode mode, and freestanding triboelectric-layer mode [40]. All these four modes share the same output equation given by Equation (1). Based on these operation modes, a large variety of TENGs have been developed for both the mechanical energy harvesting and self-powered ambient parameter monitoring/intervention [41, 42], such as micro/nanopower sources [43], blue energy [44], physical/chemical sensors [45, 46], human-machine interfaces [47, 48], nerve/muscle/brain stimulators [49, 50], air filters [51], droplet manipulation [52], and high-voltage applications [53, 54]. Hence, TENGs can contribute to be not only power sources but also various functional components in self-sustainable hybridized systems.

### 2.2. EMG

The mechanism of EMG is based on Faraday's law of electromagnetic induction, in which the voltage on a closed loop is proportionally induced by the loop's magnetic flux variation over time (*dΦ*/*dt*, where *Φ* is the magnetic flux and *t* is the time). Due to the high energy conversion efficiency, EMGs have been widely adopted in modern energy farms for centralized and large-scale electricity generation. Besides, EMGs have also been developed as distributed and small-scale energy sources for harvesting the in situ energy from various machinery vibrations and human motions [55–57]. When there are relative movements between the magnets and coils in the EMGs, induction current will be generated in the coil. According to the difference in relative movements, EMGs can be classified into two basic operation modes, i.e., movable magnet-fixed coil mode and movable coil-fixed magnet mode. Compared to TENG, EMG normally exhibits small impedance with large current but small voltage outputs. Due to these distinct output characteristics and the similar triggering forms of EMG and TENG (both by the relative movements of different components), EMG can be a good complement to TENG in a hybrid generator and hybridized system.

### 2.3. PENG

The fundamental mechanism of PENG is the appearance of an electric potential (electric dipole moment) on a piezoelectric material when it undergoes external pressure, which is also known as the direct piezoelectric effect [58–60]. The common piezoelectric materials can be categorized into two classifications—inorganic piezoelectric materials and organic piezoelectric materials [61]. The popular inorganic materials include piezoelectric ceramics and crystals, such as lead zirconate titanate (PZT), aluminum nitride (AlN), barium titanate (BaTiO_3_), lithium niobate (LiNbO_3_), zinc oxide (ZnO), and quartz. Meanwhile, the representative organic piezoelectric materials are polyvinylidene fluoride (PVDF) fiber/thin film and its copolymers, with good flexibility and suitability for wearable electronics. One of the key parameters determining the output performance of piezoelectric materials is the piezoelectric coefficient, *d*_33_ or *d*_31_, which is the generated charge density normalized by the applied stress (unit: C N^−1^). The “3” here denotes the polar axis direction of the material, and “1” can be used to denote one of the directions that are perpendicular to the polar axis due to symmetry. Thus, depending on the direction of the applied stress, PENGs can be classified into two modes, 33-mode (stress along the polar axis) and 31-mode (stress perpendicular to the polar axis). Benefitting from the advanced transferring technology in recent years, rigid piezoelectric materials with normally higher piezoelectric coefficient can also be transferred to flexible substrates, greatly improving the output performance of PENGs in wearable and implantable applications [62, 63].

### 2.4. PyENG

The operation mechanism of PyENG is based on the pyroelectric effect of a material, referring to the spontaneous polarization change under the temperature variation over time (*dT*/*dt*, where *T* is the temperature and *t* is the time) [64, 65]. Generally, the spontaneous polarization intensity of a pyroelectric material will remain unchanged (no pyroelectric current) when there are no temperature variations over time, no matter how high/low the temperature is or with/without a temperature gradient over space. Once there is an ascent or descent in temperature over time, the spontaneous polarization intensity of a pyroelectric material will then change accordingly, generating a pyroelectric current in the external circuit until a new equilibrium is achieved. Since most piezoelectric materials also exhibit pyroelectric property, thus the same material can be used to harvest both mechanical and thermal energies under different usage scenarios, based on the coexisted piezoelectric and pyroelectric property.

### 2.5. TEG

If a temperature gradient exists in a thermoelectric material, an electric potential will be built up at the two ends of the material, according to the Seebeck effect [66]. The Seebeck effect refers to the buildup of an electric potential difference across a thermoelectric material (typically semiconductor or conductor), because of the existence of a temperature gradient and the resultant diffusion of charge carriers from the hot end to the cold end. The output performance of a thermoelectric material is closely relative to its figure of merit (*ZT*) and the temperature difference (Δ*T*). The figure of merit can be given by [67]

(2) $$\begin{array}{c} ZT=\frac{S^{2}\sigma T}{\kappa }, \end{array}$$

where *S* is the Seebeck coefficient of the material, *σ* is the electrical conductivity, *T* is the absolute temperature, and *κ* is the thermal conductivity. Depending on the major charge carriers, thermoelectric materials can be divided into *n*-type (electron charge carriers) and *p*-type (hole charge carriers). Under the same temperature difference, *n*-type materials and *p*-type materials will have the built-up electric potential of opposite polarity. Thus, multiple couples of *n*-type and *p*-type thermoelectric materials can be connected in series to improve the overall output performance of a TEG.

### 2.6. SC

The underlying mechanism of SC is the photovoltaic effect, where the electron and hole pairs are generated after the absorption of lights. After the separation of the electron and hole pairs by the internal built-in electric field, an electric potential is created between the two electrodes [68]. The power conversion efficiency (*η*) is one of the key parameters to evaluate the performance of an SC, which is given by [9]

(3) $$\begin{array}{c} \eta =\frac{P_{\max}}{P_{\text{in}}}=\frac{V_{\text{oc}}\times J_{\text{sc}}\times \text{FF}}{P_{\text{in}}}\times 100\%, \end{array}$$

where *P*_max_, *P*_in_, *V*_oc_, *J*_sc_, and FF are the maximum output performance, the input solar energy, the open-circuit voltage, the short-circuit current density, and the fill factor, respectively. Nowadays, state-of-the-art SCs can reach an efficiency of more than 40% [69]. Other than the conventional rigid SCs, flexible SCs have also been developed for wearable and more diverse applications [70].

## 3. Output Enhancement Strategies for TENGs

For the TENG-based hybrid generators, the output performance of TENG components plays an important role in determining the overall output of the hybrid generators. Therefore, it is of great significance to enhance the performance of the integrated TENG components. The surface charge density of TENGs is inevitably limited by the air breakdown effect between two triboelectric surfaces and thereby is severely detrimental to their practical applications [70, 71]. As the earliest strategy to enhance performance, TENG devices are improved by material selection [72], structure optimization [73], surface modification [74], ion injection [75, 76], and environment control [77]. Charge improvement from material modifications is finite while a vacuum strategy limits applications of TENGs; therefore, more effective methods are desired to improve the charge density in air for broad applications of TENGs. There are still urgent needs for developing advanced mechanisms in enhancing the output, which should also be facile to be integrated into the practical devices for different working environments. Recently, there are a few effective methods proposed to obtain an optimum contact structure and improved output performance.

**Figure 2:**
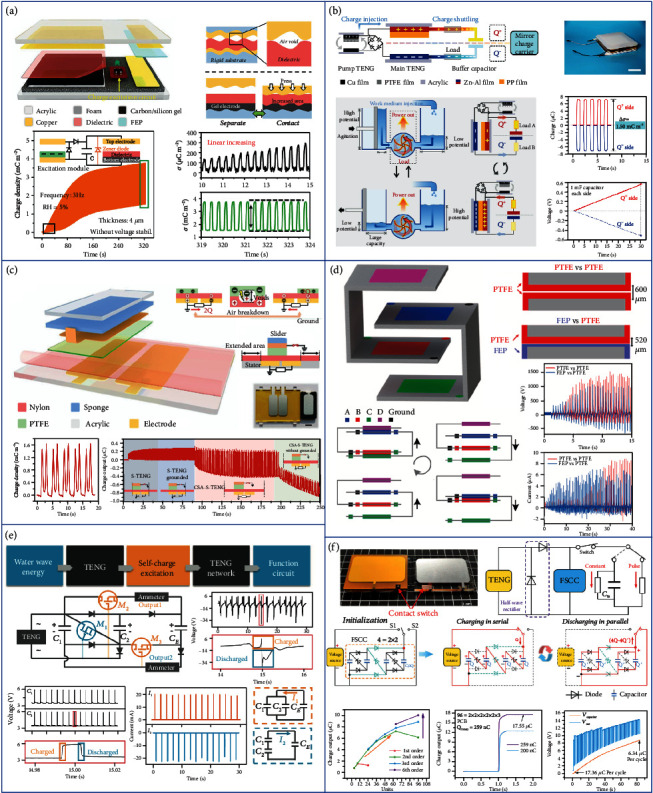
Output enhancement strategies for TENGs. (a) The air breakdown model of a charge excitation TENG to enhance charge density. Reproduced with permission from Ref. [78], copyright 2020 Springer Nature. (b) A high-performance TENG based on charge shuttling. Reproduced with permission from Ref. [79], copyright 2020 Springer Nature. (c) Boosting the output performance of a sliding mode TENG by charge space accumulation effect. Reproduced with permission from Ref. [82], copyright 2020 Springer Nature. (d) The out-of-plane design of a Bennet doubler-based TENG. Reproduced with permission from Ref. [83], copyright 2020 Elsevier B.V. (e) A high-output charge excitation TENG based on the voltage-multiplying circuit. Reproduced with permission from Ref. [86], copyright 2020 Wiley-VCH. (f) The switched capacitor convertors based on the fractal design for TENG output enhancement. Reproduced with permission from Ref. [87], copyright 2020 Springer Nature.

Liu et al. proposed a standard method to precisely evaluate the contact status of TENG to optimize the contact of two tribo-surfaces, as shown in Figure 2(a) [78]. They illuminate the strategies of enhancing the charge output for the charge excitation TENG, including the reduction of the thickness of dielectrics, the increment of external capacitor, and the control of the atmospheric environment. With the quantified contact, an arched composite soft structure is designed by using a homemade carbon/silicone gel electrode, which can enhance the contact efficiency of devices from 6.16% to 54.98% for a 4 *μ*m dielectric film. As a result, the average charge and the energy density for the TENG with charge excitation are achieved up to 2.38 mC m^−2^ and 286.7 mJ m^−2^, respectively, in the ambient atmosphere with 5% relative humidity. Furthermore, the experimental result indicates that the actual charge density in the electrode is over 4.0 mC m^−2^, showing a large possibility for further promotion of output charge density using this method.

As illustrated in Figure 2(b), Wang et al. proposed a high-performance TENG based on the shuttling of charges [79]. The charge shuttling TENG consists of a pump TENG, a main TENG, and a buffer capacitor. The electrodes of the main TENG and the buffer capacitor form two conduction domains, presenting a quasisymmetrical structure with a *Q*^+^ side and a *Q*^−^ side. The capacitance of the main TENG changes upon contacts and separations, while that of the buffer capacitor remains constant, inducing voltage differences between them. Therefore, the charges would be shuttled between the main TENG and the buffer capacitor in a quasisymmetrical way, generating electricity on the two loads. When the main TENG changes to the contact state, the capacitance of the main TENG grows, causing the voltage on it to descend. Therefore, charges return from the buffer capacitor to the main TENG via the loads. Consequently, an ultrahigh projected charge density of 1.85 mC m^−2^ is obtained in the ambient conditions. Based on this mechanism, an integrated device for water wave energy harvesting shows the feasibility of the charge shuttling TENG as a fundamental device to be applied in complex structures for various practical applications.

TENG in the lateral sliding mode provides an effective approach for the in-plane low-frequency mechanical energy harvesting. However, as the output enhancement strategies such as surface modification and charge excitation are not well applicable for this mode, the strategy to promote its output performance is rarely proposed [80, 81]. As shown in Figure 2(c), He et al. developed a new strategy by a shielding layer and alternative blank-tribo-area-enabled charge space accumulation (CSA) design for enormously improving the charge density of a sliding mode TENG [82]. The CSA-TENG consists of polytetrafluoroethylene (PTFE) and a nylon (PA) film, which also incorporates a shielding layer on the slider and an alternating blank-tribo-area structure on the stator. With the grounded conductive layer covered on the back of the slider, air breakdown can be contained to a great extent, and by further introducing the extra blank-tribo-area structure with charge-dissipated tribo-material on the stator, the CSA effect can be achieved. The CSA process can be clearly observed in the charge output with a fast increment to a stable state, which is consistent with the theoretical analysis. With experimental optimization, the stable output charge density reaches 1.63 mC m^−2^. Additionally, when the grounding state of CSA-TENG is cut off, a rapid decline in the charge output is observed, proving the inhibitory effect on the air breakdown of the shielding electrode. In addition, based on the principle of the linearly sliding TENG unit, CSA-TENG can be easily designed to a rotational working mode.

Similarly, based on the CSA mechanism, Wang et al. presented an out-of-plane design to achieve high-performance TENG [83]. As shown in Figure 2(d), electrodes B and D are arranged on the left part while electrodes A and C are arranged on the right part of the designed structure. There are two different dielectric combination groups, i.e., PTFE vs. PTFE with 600 *μ*m thickness and FEP vs. PTFE with 520 *μ*m thickness. Both groups can generate the CSA to enhance the output performance. Since the friction material is the same for the PTFE vs. PTFE group, the voltage increases slower than that of the FEP vs. PTFE group. However, due to a thicker dielectric layer, the PTFE vs. PTFE group shows a higher maximum voltage. The maximum voltage is 1400 V and 1150 V for the PTFE vs. PTFE and the FEP vs. PTFE group, respectively. Both two groups reach the maximum peak power at around 200 M*Ω* with 3.3 and 2.5 mW, respectively.

Another common method for increasing the output of TENGs is designing the external circuit. Owing to the advantages of easy integration and being magnet-free and lightweight, the switched capacitor convertor (SCC) plays an increasingly important role compared to the traditional transformer in some specific power supply systems [84, 85]. Therefore, a power management system with higher transfer efficiency and multifunctional output mode is urgently needed and has great significance for practical applications of TENGs. As shown in Figure 2(e), Liang et al. developed a new charge excitation system based on the voltage-multiplying circuit (VMC) to achieve high-output TENGs for effectively harvesting the water wave energy [86]. Not only the output performance of a single TENG is increased by multiple times but also a scheme is proposed to realize a high-output TENG network through integrating with the charge excitation circuits (CECs). When connecting with the CEC, the AC outputs of a TENG can be increased by many times and transformed into DC outputs simultaneously. When triggered by real water waves, the outputs of the charge excitation TENG are found to be controlled by the water wave frequency and amplitude. Under the optimal water wave condition with a frequency of 0.6 Hz and an amplitude of 10 cm, the output current and power can reach the maximum values of 25.1 mA and 25.8 mW, respectively. Furthermore, a TENG network integrated with the CECs is proposed and fabricated to harvest the water wave energy, presenting a maximum output current of 24.5 mA and a power of 24.6 mW. The CEC can improve both the output current and power of a TENG, which is the most important difference from an ordinary voltage doubler circuit that only increases the output voltage. Finally, the high-output TENG network is utilized to drive a thermometer to work continuously and realize wireless communication with a mobile phone for remote environmental monitoring.

However, the high output impedance and switching loss largely reduce the SCC's power efficiency, due to the imperfect topology and transistors. To address this issue, as shown in Figure 2(f), Liu et al. proposed fractal design-based switched capacitor convertors (FSCC) with characteristics including high conversion efficiency, minimum output impedance, and electrostatic voltage applicability [87]. Considering the low charges (~200 nC) in TENG, large switching loss, and zero gate voltage drain current (>15 *μ*A) of MOSFET and superlow leakage current (<1 nA) of the rectifier diode, the SCC composed of rectifier diodes and capacitors is designed to convert the electrostatic voltage of TENG. However, with the increasing stages of diodes as switches, the basic SCC will inevitably have large output voltage drop loss caused by diodes during the discharging process, leading to low energy conversion efficiency. The FSCC provides significant guidance for the development of power management toward multifunctional output in numerous applications. Fractal means that a rough or piecemeal geometric shape can be divided into several parts, and each part is reduced and has the self-similar property. By integrating the FSCC power management system on a printing circuit board (PCB), over 67 times charge boosting, 14.3 A m^−2^ current density, and 954 W m^−2^ power density can be reached by a common TENG under a pulse output, for driving electric devices like a buzzer. Under a constant output mode, over 94% of total energy transfer efficiency is realized with an output power density of 37.09 mW m^−2^, and mobile electric devices like digital Vernier caliper and temperature hygrometer can be driven continuously by the TENG with the FSCC power management system.

## 4. TENG-Based Hybrid Generators for Outdoor Applications

Nowadays, the energy source in our life is highly dependent on the usage of fossil fuels. However, the total amount of fossil fuels is limited and the pollution issue caused by fossil fuels is more and more serious. Thus, the clean and renewable energy sources existing in the ambient environment, e.g., mechanical, thermal, and solar energy, are in urgent need to be utilized to solve the oil shortage issues, as well as benefit the sustainable development of the earth. Among these clean energy sources in the outdoor environment, mechanical energy is highly ubiquitous and abundant that exists in various forms such as ocean, wind, and vibration.

Considering that 70% of the earth is covered by water, water energy exhibits advantages of wide universality and easy accessibility. Previously, the most common method for water energy harvesting is using EMG to convert the water energy into electric power by a turbine, but this kind of method means low energy conversion efficiency, large device volume, and high cost for equipment maintenance. Considering that the optimal operation frequency of EMG is higher than 50 Hz, not all water motions are suitable to be harvested by EMG, especially for those rectilinear motions operating below 10 Hz, i.e., wave energy. In this regard, TENGs that are capable of scavenging low-frequency (<5 Hz) mechanical energy with the advantages of low cost, high voltage, and simple fabrication have been explored globally in recent years for low-frequency wave energy harvesting [88–92]. Moreover, the energy conversion efficiency of TENG can be further improved by integrating with EMG to form a hybrid generator, where the low output current of TENG can be compensated by the high output current of EMG, and the unsatisfactory performance of EMG in the low-frequency energy scavenging can also be made up by TENG [93, 94]. Here, several latest TENG-EMG hybrid generators for wave energy scavenging are summarized and discussed.

**Figure 3:**
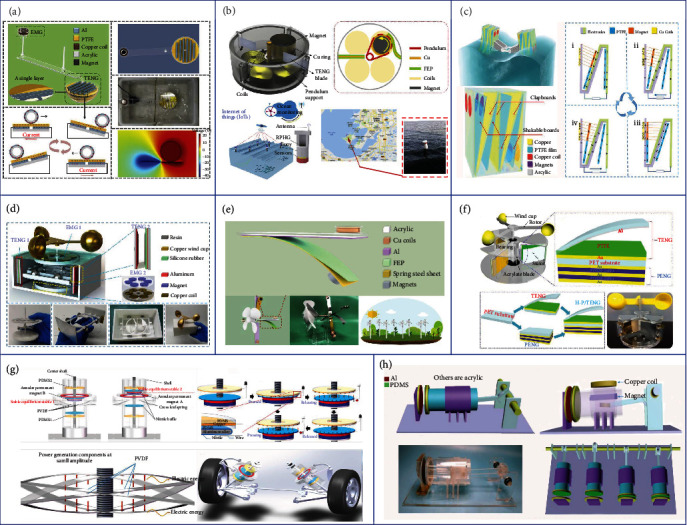
TENG-based hybrid generators for outdoor mechanical energy harvesting. (a) A teeterboard-like TENG-EMG hybrid generator for harvesting low-frequency ocean wave energy. Reproduced with permission from Ref. [95], copyright 2019 Elsevier B.V. (b) A rotational pendulum-based TENG-EMG hybrid generator designed for ultralow-frequency blue energy harvesting. Reproduced with permission from Ref. [96], copyright 2019 Elsevier B.V. (c) A double-swing TENG-EMG hybrid generator for highly efficient wave energy harvesting. Reproduced with permission from Ref. [73], copyright 2019 Wiley-VCH. (d) A TENG-EMG hybrid generator with contact-separation mode TENG for low-frequency wind energy harvesting. Reproduced with permission from Ref. [103], copyright 2019 Elsevier B.V. (e) A windmill-like TENG-EMG hybrid generator for steady and efficient energy harvesting of low-speed wind. Reproduced with permission from Ref. [104], copyright 2020 Springer Nature. (f) A rotational TENG-PENG hybrid generator for highly efficient and stable wind energy harvesting. Reproduced with permission from Ref. [107], copyright 2018 Elsevier B.V. (g) A bistable broadband TENG-PENG hybrid generator for energy harvesting of ambient low-frequency rectilinear motions. Reproduced with permission from Ref. [114], copyright 2019 Elsevier B.V. (h) A crankshaft piston-based TENG-EMG hybrid generator for rotational mechanical energy harvesting. Reproduced with permission from Ref. [115], copyright 2018 Wiley-VCH.

As illustrated in Figure 3(a), a teeterboard-like hybrid nanogenerator was developed by Wu et al. with a multilayered TENG floating on water at one end and an EMG driven by a lever which moved vertically at the other end [95]. With the novel separate design, the lightweight TENG end can be easily triggered by low-frequency ocean waves, resulting in the freestanding Al tubes rolling on the PTFE surface and generating desirable triboelectric outputs. Besides, through the unique lever design, the working amplitude of the EMG is significantly increased, solving the issue of low efficiency of EMG at low frequencies to a certain extent. At the optimum working condition, the maximum voltage of the TENG and the maximum current of the EMG can reach 760 V and 10 mA, respectively. By integrating the output from both TENG and EMG with rectifier circuits, a raindrop sensor can be directly driven by the hybrid generator under simulated tide flow, showing the potential of this device in large-scale wave energy harvesting applications. A rotational pendulum-based TENG-EMG hybrid system was proposed by Hou et al. as shown in Figure 3(b) [96]. The unrestricted rotational movement of the pendulum enables the hybrid generator with wide applicability to low-frequency (<5 Hz) and irregular vibration. When the pendulum rotor rotates, the magnetic flux across each coil will change and generate the electromagnetic output. Besides, the Cu ring settled on the rotor will also contact with the PTFE intermittently during rotating, thus generating the triboelectric output. After configuration optimization, the maximum power density of the TENG and EMG can reach 3.25 W m^−2^ and 79.9 W m^−2^, respectively, with the water wave frequency of 2 Hz and amplitude of 14 cm. Combining these two outputs, the hybrid generator is successfully demonstrated to be integrated into a buoy which is able to utilize the energy from waves to directly drive IoT sensors. Different from sliding or rolling freestanding mode-based TENGs that always move together with the water waves and transform the limited wave energy, a TENG-EMG hybrid generator with a cubic structure which works in contact freestanding mode was reported by Wang et al. to further improve the energy conversion efficiency, as shown in Figure 3(c) [73]. The external oscillation enables the reciprocating contact and separation of the PTFE board between two electrodes, altering the electrodes' potential difference and thus generating the triboelectric output. At the same time, the magnets embedded with the PTFE board move toward or away from the coils, creating the electromagnetic current which synchronizes with the TENG output. After being installed on the one side of a wing-structure bracket for wave energy harvesting, the TENG part of the hybrid generator can reach a maximum *V*_oc_ of 400 V and *I*_sc_ of 15.3 *μ*A with a wave frequency of 1 Hz, and the EMG part is able to achieve a *V*_oc_ of 1.7 V and *I*_sc_ of 5.4 mA. These two complementary outputs greatly enhance the total energy harvesting efficiency after parallel connection, which are then successfully demonstrated to drive a thermometer, showing its prospect for future large-scale blue energy harvesting.

Apart from the ubiquitous wave energy, wind energy that is abundant on land is another reliable energy source to be utilized for solving the energy issues, as well as providing sustainable power for sensors under the IoT framework [97–100]. In order to efficiently harvest the wind energy under different frequencies, the combination of TENG and EMG has been explored and proven as an effective approach with high energy conversion efficiency benefitting from the complementary properties of the two energy harvesting mechanisms. However, most triboelectric-integrated wind energy harvesters are based on rotational structures, resulting in inevitable abrasion between two friction layers and reduction in device life [101, 102]. One way to solve this problem is to change the working mode of TENG from being sliding based to contact-separation based. As shown in Figure 3(d), a TENG-EMG hybrid wind generator was proposed by Fan et al. [103]. For the EMG part, the output is generated by the relative rotating motion between the upper magnet rotator and the bottom coil stator. For the TENG part, the novel structural design of the slider can help to convert the rotating motion of the rotator into the driving force of its own reciprocating motion, thus resulting in the contact and separation between the aluminum and the silicone rubber for the triboelectric output generation. With a wind speed of 9 m s^−1^, the maximum output power of TENG and EMG could reach 0.36 mW and 18.6 mW, respectively. By connecting the hybrid generator with a rectifier circuit and a storage capacitor, a hygrothermograph can be directly powered to transmit weather-related data wirelessly to the server. Although the friction of the TENG part has been reduced by using the contact-separation mode, friction still exists at the rotational EMG which requires the slowest driving wind speed to exceed 4 m s^−1^, greatly limiting its application for low-speed breeze energy harvesting. Thus, Zhang et al. reported a windmill-like hybrid generator applicable to low-speed wind as illustrated in Figure 3(e) [104]. The spring steel sheet and the magnets mounted on the fan can effectively reduce the rotation resistance. On the one hand, the spring steel sheet electrodes and the magnets are the essential components of TENG and EMG, respectively. On the other hand, the spring steel sheet can store the elastic potential energy during rotation and convert it into kinetic energy to enhance the contact-separation motion of TENG, while the weight of the magnets can also help to weaken the electrostatic adsorption between the triboelectric layers, leading to the device successfully driven at a low wind speed of 1.8 m s^−1^. With matched loads, the output power of TENG and EMG could reach 0.95 mW and 3.7 mW, respectively, for powering electronic devices. Other than the TENG-EMG hybrid generator, PENG is another suitable mechanism to be easily hybridized with TENG due to the similar thin-film nature of their active layers [105, 106]. Besides, the wide material selection of PENG and TENG reduces the difficulty of adjusting matching impedance, which could help to avoid extra energy loss brought by transformers. Zhao et al. reported a TENG-PENG hybrid nanogenerator for wind energy harvesting as shown in Figure 3(f) [107]. The rotator will be driven to rotate by wind and force the polyethylene terephthalate (PET)/Al sheet to contact with the PTFE surface, thus generating triboelectric output in the Al/Au electrodes. When the PTFE film fully contacts with the Al layer, the further rotation will induce tensile stress on the integrated PENG and a 31-mode piezoelectric potential is then produced in the PVDF-TrFE layer. The integrated output of TENG and PENG could reach a high output performance of 150 V and 150 *μ*A at a wind speed of 14 m s^−1^, which is capable of powering 50 LEDs and shows the potential for future large-scale wind energy harvesting.

Except for the mechanical energy contained among water wave and wind in nature, the vibration generated by machinery and human activities, e.g., suspension systems in vehicles and human walking, can also be harvested for specific applications to avoid energy waste [108–113]. A broadband TENG-PENG hybrid nanogenerator with a bistable structure was developed by Deng et al. for ultralow-frequency rectilinear vibration as illustrated in Figure 3(g) [114]. Normally, magnet B mounted on the moveable center shaft is repelled by magnet A fixed on the shell. The center of the cross leaf spring is also fixed on the movable shaft, with four ends supported by the shell. Due to the repulsive force of the paired magnets and the cross spring's elastic force, the movable center shaft and magnet B will experience a nonlinear bistable reciprocating motion under the external excitations. This arouses the deformation of the PVDF film attached on the cross leaf spring and the contact-separation motion between the polydimethylsiloxane (PDMS) and the nitrile baffle, leading to the piezoelectric and triboelectric outputs. These two outputs compensate for each other, where the triboelectric outputs dominate when the external excitation is large enough to ensure the contact-separation motion of triboelectric layers, and the piezoelectric outputs are crucial when the external excitation is small. This device is successfully demonstrated to charge a 10 *μ*F capacitor to 0.12 V within 60 s under the excitation frequency of 0.1 Hz, proving its applicability to ultralow-frequency motions. By integrating into the suspension system of vehicles, the hybrid nanogenerator can be utilized to harvest the vibration energy of cars, which can be further stored to power electronics to reduce fuel consumption. Another hybrid nanogenerator that could be used to scavenge the energy of vehicles was reported by Yang et al. as shown in Figure 3(h) [115]. The crankshaft piston-based TENG-PENG hybrid nanogenerator could be mounted on the shafts of cars and driven by shafts' rotation. The rotational motion of the crankshaft drives the contact-separation motion between the Al and the triboelectric layers, as well as the relative motion between the magnet and copper, for generating the triboelectric and electromagnetic outputs. The maximum output power of the TENG and EMG could reach 0.08 mW cm^−2^ and 0.0295 mW, respectively, with a rotational speed of 300 rpm. These two outputs are further hybridized to drive several low-power electronics, showing its feasibility for vehicle-related rotation energy harvesting.

**Figure 4:**
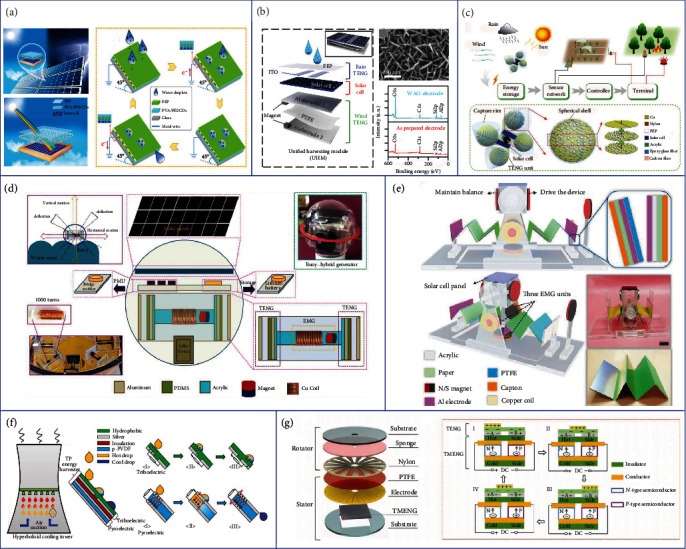
TENG-based hybrid generators for outdoor multitype mechanical/solar/thermal energy harvesting. (a) A triboelectric-photovoltaic hybrid cell for raindrop and solar energy harvesting. Reproduced with permission from Ref. [126], copyright 2020 American Chemical Society. (b) A triboelectric-photovoltaic unified module for simultaneous raindrop, solar, and wind energy harvesting. Reproduced with permission from Ref. [127], copyright 2020 Elsevier B.V. (c) A triboelectric-photovoltaic hybrid all-in-one power source for simultaneous raindrop, solar, and wind energy harvesting. Reproduced with permission from Ref. [128], copyright 2020 Wiley-VCH. (d) A fully packed spheroidal triboelectric-electromagnetic-photovoltaic hybrid generator for wave/solar energy harvesting and self-powered position tracking. Reproduced with permission from Ref. [129], copyright 2019 Elsevier B.V. (e) A nonencapsulated pendulum-like paper-based triboelectric-electromagnetic-photovoltaic hybrid generator for wave/solar energy harvesting. Reproduced with permission from Ref. [130], copyright 2019 Wiley-VCH. (f) A triboelectric-pyroelectric hybrid generator for recovering energy from low-grade waste fluids. Reproduced with permission from Ref. [132], copyright 2020 Elsevier B.V. (g) A triboelectric-thermoelectric hybrid nanogenerator for ambient mechanical energy and friction-induced heat energy harvesting. Reproduced with permission from Ref. [133], copyright 2018 Wiley-VCH.

In addition to the ubiquitous mechanical energies, solar and thermal energies are also clean and abundant energy sources in nature that can be utilized. Solar energy has been widely scavenged by using SCs in recent years [116–118]. However, the performance of SC is greatly affected by the weather such as rainy or cloudy days when there is no sunlight. A feasible strategy for this problem is to hybridize SCs with other energy harvesting devices to achieve continuous energy harvesting in varying weather conditions. TENG has been proven as a reliable energy harvester for raindrop energy scavenging based on the mechanism of triboelectrification at the liquid/solid interfaces [119–122]. Hence, TENG can be further integrated with SCs as a complement to insufficient solar energy on rainy days. Though several works have successfully demonstrated the feasibility of such kind of hybridization [123–125], the insufficient transparency of TENG top layers and the high cost of antireflective process limit their applications for efficient and large-scale energy harvesting. To address this issue, Wang et al. proposed a hybrid cell consisting of a silicone SC and a TENG with a highly transparent ionic conductor using a carbon dot composite film, as illustrated in Figure 4(a) [126]. The transmittance of the TENG top layer is significantly improved in the hybrid generator, thereby increasing SC's short-circuit current density and power conversion efficiency (from 13.6% to 14.6%). Besides, the TENG part can harvest the raindrop energy due to the triboelectrification induced on the fluorinated ethylene propylene (FEP) surface with a maximum output power of 13.9 *μ*W. The hybrid generator provides a promising method to simultaneously scavenge solar energy and raindrop energy with high power conversion efficiency. Apart from the raindrop energy, the combination strategy of solar and wind energy is also meaningful considering that there is always strong wind on rainy days. As shown in Figure 4(b), an ultrathin hybrid generator capable of harvesting raindrop, wind, and solar energies in varying weather conditions was developed by Roh et al. [127]. The hybrid device is composed of two TENGs and one SC. The top TENG consisting of two indium tin oxide (ITO) electrodes and a transparent FEP film is utilized for raindrop energy harvesting based on the triboelectrification on the FEP surface and electric potential changes in the two ITO electrodes when the droplets slide over the FEP film. Besides, the high transparency of both the ITO and FEP layers allows sufficient sunlight to reach the SC to avoid too much light absorption by top TENG layers. The thin PTFE film of the bottom TENG will vibrate with the incoming wind blows, resulting in an alternative contact-separation motion between the PTFE film and two Al electrodes for triboelectric output generation. The maximum output voltage of the rain TENG and the wind TENG could reach 5 V and 50 V, respectively, which is able to charge a 0.1 *μ*F capacitor to 14 V within 14 s. At the same time, the SC can provide an output voltage of 4.2 V, showing the potential of using the hybrid generator for effective energy harvesting in varying weather conditions. Another novel all-in-one hybrid generator for wind, raindrop, and sunlight energy harvesting was proposed by Xu et al., as depicted in Figure 4(c) [128]. The hybrid generator consists of four spherical TENG units covered by truncated cone-shaped capture rims for wind and rain fluid energy harvesting and four SCs mounted on two sides of the acrylic frame for solar energy collecting. In each TENG unit, there are multiple disks attached with Cu electrodes sealed in the shell with a 5 mm space where the Cu electrodes on the same hemisphere side are connected together to form two total outputs. FEP pellets are filled in the spaces to serve as the negative triboelectric material. When the spherical shell is driven to rotate by external excitation, the FEP pellets will always contact with the electrode that is currently located at the bottom of the shell due to gravity, making the pellets contact and separate alternately between the two electrodes and generating a high average triboelectric output power of nearly 5.63 mW. Combining the outputs from TENG units and SCs, several sensors can be successfully powered by the hybrid generator to implement sustaining self-powered soil moisture control, forest fire prevention, and pipeline monitoring for all-weather applicable IoT applications.

The above works utilize TENGs to scavenge the weather-related mechanical energies to complement the output of SCs from solar energy, forming the hybridization of two energy harvesting mechanisms. To further improve the energy harvesting efficiency, TENG, EMG, and SC can be integrated together to form a tri-mechanism-based hybrid generator with the merits of enhanced energy scavenging ability for mechanical energies brought by the complementary properties of TENG and EMG as mentioned previously. Chandrasekhar et al. reported a fully packed spheroidal hybrid generator consisting of a TENG and an EMG for water wave energy harvesting and a solar panel as a backup power source for low wave condition, as illustrated in Figure 4(d) [129]. When the hybrid generator is driven to sway by the water wave, the cylinder tube will slide back and forth in the device shell, resulting in the alternative contact and separation between the PDMS and the Al layers of two TENGs and producing the triboelectric output. In this process, the magnet placed in the tube will also slide across the coils wrapped around the tube, inducing magnetic flux changes and generating electromagnetic currents. The maximum outputs of the TENG and EMG could reach 100 V/2 *μ*A and 20 V/15 mA, respectively, which are able to be integrated by bridge rectifiers and further hybridized with the SC's output to charge a lithium battery and power a position-tracking long-range device for self-powered ocean-based tracking application. Based on the same trimechanism hybrid strategy, a nonencapsulated pendulum-like paper-based hybrid generator was developed by Yang et al., as shown in Figure 4(e) [130]. There are three components in this device: one solar panel on the mover's top, two paper-based multilayered TENG units, and three EMG units. One of the two magnets mounted on the top portion of the mover is used to drive the device to wobble based on the repulsive force between magnets when varying magnetic field is induced by water waves, and the other one is used to maintain the balance of the device. During the pendulum-like motion of the mover, continuous contact-separation motions of the PTFE and Al layers in TENG units, as well as the change of magnetic flux in copper coils, will be triggered to generate the electrical output, where the maximum output power of the TENG and EMG units could reach 22.5 mW and 1.39 mW, respectively. By integrating an SC for additional energy harvesting, the volume of the device could be effectively utilized, presenting a new strategy of hybrid generators to realize multisource energy harvesting for a self-powered navigation system.

Besides solar energy, thermal energy is another valuable static energy in the outdoor environment that is also desirable to be harvested considering the wide distribution of heat resources existing in geothermal and industrial factories, e.g., power plants, chemical plants, and metal productions [131]. Moreover, most thermal energy appears in the form of hot water, which contains not only the heat but also the kinetic energy. TENGs have been proven as reliable energy harvesters to scavenge the kinetic energy from water drops based on the solid-liquid triboelectrification, so it is a good strategy to hybridize PyENGs or TEGs with TENGs to fully utilize both the thermal and kinetic energies for maximum energy conversion efficiency. Jiang et al. proposed a TENG-PyENG hybrid generator aiming at collecting the energy from low-grade waste fluids, as shown in Figure 4(f) [132]. When a hot droplet slides across the surface of the hydrophobic layer, an electric potential difference in the attached two Ag electrodes arises, thus producing the triboelectric output. Meanwhile, the heat of the hot water is transferred to the PVDF layer, resulting in the oscillation of dipoles and the change of polarization in the PVDF film, thus generating the pyroelectric output. By combining these two outputs with rectifier circuits, a maximum output power of 2.6 *μ*W cm^−2^ could be achieved with a 238% energy increment compared with a pure thermal energy harvesting strategy. In addition to scavenging the existing thermal energy in the ambient environment, huge heat energy will be produced during the friction process of TENGs when harvesting mechanical energies. This friction-induced heat can also be further utilized to reduce energy loss and increase energy conversion efficiency. As illustrated in Figure 4(g), Wu et al. integrated a TEG into a 2D rotary TENG to harvest the extra thermal energy induced by the TENG's friction [133]. The triboelectric output is generated by the relative friction between the stator and rotator, while the thermoelectric output is produced because of the temperature difference between the friction-induced hot side and the bottom cold side. By connecting the TENG and the TEG in series, the hybrid generator could produce a *V*_oc_ of 4.6 V and an *I*_sc_ of 0.4 mA at a rotation rate of 500 rpm, which are much higher than the outputs generated by the pure TENG (*V*_oc_ of 2.5 V and *I*_sc_ of 0.4 mA), showing the significant complementary effect of TEG on enhancing TENG's energy conversion efficiency.

## 5. TENG-Based Hybrid Generators for Indoor Applications

With the rapid development of the IoT-enabled smart home systems, increasing smart electronics can be found in our daily lives in the indoor environment. Though batteries can provide a reliable power supply for most of these smart devices, their lifetime is limited and the recharging process is also labor-intensive. Besides, the extensive use of batteries will also cause severe pollution to the environment and increase power consumption. In recent years, advanced energy harvesting technologies have been developed to effectively scavenge the indoor energies, e.g., biomechanical energy from human activities [134, 135], waste heat energy from appliances [136], and indoor light [137], to directly power smart electronic devices and realize sustainable systems without too much maintenance operations. TENG, due to its wide selection of materials and high energy conversion efficiency for low-frequency excitation, is quite suitable to be designed as different household products, e.g., floor [138–142] and bed sheet [143], and wearable devices [13, 22, 144–146] for indoor biomechanical energy harvesting and sensing. Moreover, by integrating TENGs with other energy harvesting mechanisms such as EMG, PENG, PyENG, TEG, and SC, hybrid generators could be implemented toward effective indoor energy harvesting applications.

**Figure 5:**
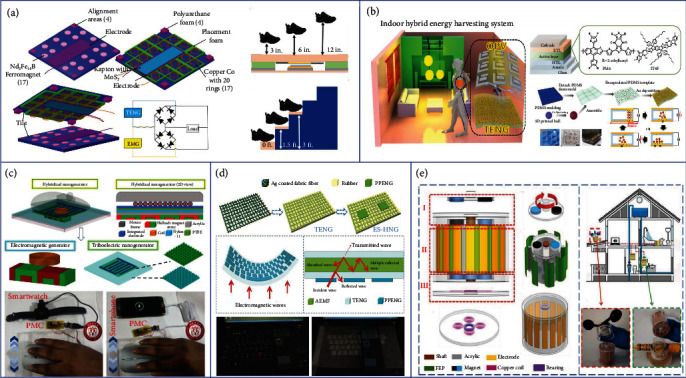
TENG-based hybrid generators for indoor energy harvesting. (a) A triboelectric-electromagnetic hybrid energy tile for biomechanical energy harvesting. Reproduced with permission from Ref. [147], copyright 2020 Elsevier B.V. (b) A triboelectric-photovoltaic hybrid smart floor for simultaneous biomechanical and indoor light energy harvesting. Reproduced with permission from Ref. [148], copyright 2020 Elsevier B.V. (c) A human-machine interactive triboelectric-electromagnetic hybrid mouse-like generator for biomechanical energy harvesting. Reproduced with permission from Ref. [149], copyright 2020 Elsevier B.V. (d) A triboelectric-piezoelectric-pyroelectric hybrid keyboard cover for typing-related energy harvesting. Reproduced with permission from Ref. [150], copyright 2018 Wiley-VCH. (e) A triboelectric-electromagnetic hybrid generator driven by magnetic coupling for fluid energy harvesting and self-powered flow monitoring. Reproduced with permission from Ref. [151], copyright 2019 Wiley-VCH.

As shown in Figure 5(a), Islam et al. reported a triboelectric-electromagnetic hybrid energy tile for stepping energy harvesting [147]. The TENG part is composed of a layer of Al and a layer of high-polarized Kapton serving as the two opposite triboelectric materials, and the EMG part consists of multiple pairs of coils and magnets. When the smart tile is compressed by human footsteps, there will be contact-separation motions induced between the Kapton and the Al layer, as well as relative vertical motions in the coil and magnet pairs, thus producing the triboelectric and electromagnetic outputs. With rectifier circuits for further integration, the maximum output power of this hybrid tile could reach 6 W, showing its feasibility for sustainable indoor energy harvesting. By further integrating an organic photovoltaic cell (OPVC) for indoor light energy collection, a TENG-OPVC hybrid floor was proposed by Jung et al., as indicated in Figure 5(b) [148]. High power conversion efficiency (15.03%-16.45%) could be achieved with an optimal OPVC for indoor light conditions. Besides, the mechanical shaking induced by human walking will drive the Cu balls to contact and separate with the inner surface of the PDMS within the hollow cuboids, generating a triboelectric power of higher than 3 *μ*W. The hybrid generator is successfully demonstrated to charge a lithium-ion battery with high speed and efficiency, illustrating its potential for powering indoor electronics.

In the information age, the usage of computers has become an indispensable part of our daily activities, where the biomechanical energies generated from hand motions could also be utilized for self-powered smart electronic purpose. A TENG-EMG hybrid smart mouse was developed by Rana et al., as depicted in Figure 5(c) [149]. The TENG installed in the smart mouse consists of two parts: a sliding part composed of a PTFE film with two pairs of interdigitated electrodes and an immobile part made up of the nylon-11 film as the mouse pad. When the mouse slides on the mouse pad, the electric potential in the electrode pairs will change, thus driving the electrons to flow and generating the triboelectric output. In this process, the relative motion between the coils mounted in the mouse and the Halbach magnet fixed underneath the pad will also induce the continuous magnetic flux change in the coils and generate the electromagnetic output. This hybrid generator demonstrates a maximum output power density of 185 W m^−2^ by scavenging the biomechanical energy of hand motions. The energy is successfully utilized to power portable electronics including a Bluetooth mouse, smartwatches, and smartphones. In addition to the mouse, the keyboard is also a commonly used human-computer interaction device, which can be combined with a hybrid generator to collect the mechanical energy of finger tapping. A triboelectric-pyroelectric-piezoelectric hybrid nanogenerator designed as the computer keyboard cover was proposed by Zhang et al., as shown in Figure 5(d) [150]. The top and bottom rubber layers serve as the negative triboelectric material in the device, and the triboelectric output will be produced in the embedded Ag-coated fabric fiber when the finger taps on the rubber surface. The pressure of finger tapping induces piezoelectric polarization charges of the pyroelectric-piezoelectric nanogenerator (PPENG), which is more sensitive to strain compared with the TENG unit. Besides, the PPENG can also harvest the thermal energy of the human body based on the pyroelectric effect to enhance the output performance of the hybrid nanogenerator. The hybrid nanogenerator is able to charge a capacitor to 3 V within 200 s by tapping, which can be directly used to power portable devices.

Using the same generator to harvest the outdoor wind energy and the indoor water flow energy simultaneously is also a good strategy to realize sustainable smart home systems. As illustrated in Figure 5(e) [151], a TENG-EMG hybrid generator was proposed by Zhong et al. for collecting energies from multiple sources. The proposed hybrid generator consists of a cylindrical stator with multiple attached electrodes and a cylindrical rotator with several FEP films mounted on the outer surface. When the top driver of the device is driven to rotate by wind or water flow, the rotator will spin due to the magnetic coupling force, resulting in the contact-separation motion between the FEP film blades and the fixed Cu electrodes for triboelectric output generation. Meanwhile, the magnets in the bottom of the rotating rotator will also induce electromagnetic output in the copper coils fixed underneath the lower stator. With a rotating speed of 500 rpm, the maximum output power of the TENG and the EMG could reach 1.05 mW and 58.3 mW, respectively. The combined output power is sufficient to directly drive small electronics, e.g., humidity sensor and thermometer, indicating that the practical issues of sustainable power supply in certain applications could be solved by such hybridizing strategy for smart home, smart building, and smart city.

## 6. TENG-Based Hybrid Generators on Human Bodies

Along with the rapid development of TENG-based hybrid generators for outdoor and indoor applications, they are also extensively explored in different aspects of wearable and implantable applications, such as sustainable power sources, self-powered sensors, human-machine interfaces, and healthcare monitoring.

### 6.1. Wearable Hybrid Generators

Due to the capability of converting mechanical energy into electricity, wearable TENGs are suitable for acting as energy harvesters and self-powered sensors, especially to detect human daily activities and physiological status [42, 47, 83, 152–156]. Even though TENGs hold great promise as wearable electronics, they still inevitably have limitations in power generation, sensing range, sensitivity, and also the sensing domain for the intrinsic limitations of electrification [157–159]. Therefore, hybrid generator systems that combine multiple energy harvesting units with energy storage units are widely studied for wearable electronics.

**Figure 6:**
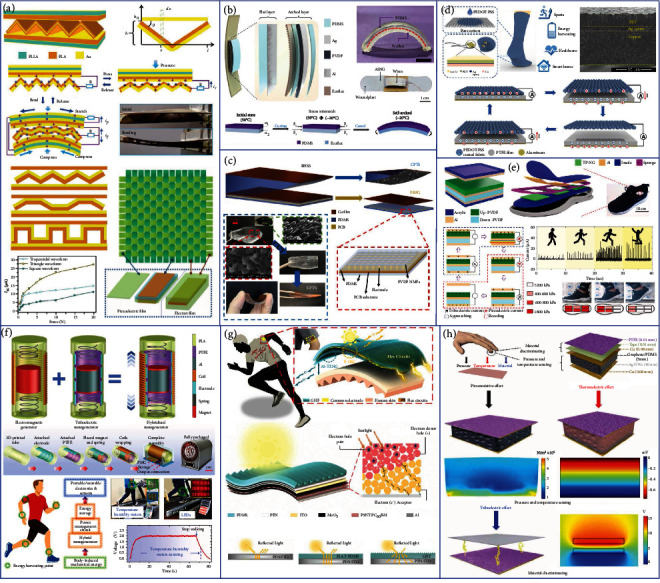
TENG-based hybrid generators on the human body for wearable electronics. (a) A nonpiezoelectric meso-PLA electret-based TENG combined with a double-layered PLLA-based PENG for E-skin application. Reproduced with permission from Ref. [109], copyright 2020 Wiley-VCH. (b) A self-powered pulse sensor based on a self-arched structure with a combined effect of a PENG and a TENG. Reproduced with permission from Ref. [163], copyright 2020 Elsevier B.V. (c) A biomimetic and flexible self-powered sensor with hybridized TENG and PENG to enhance the energy harvesting characteristics. Reproduced with permission from Ref. [164], copyright 2020 Elsevier B.V. (d) A self-powered and self-functional cotton sock based on the piezoelectric and triboelectric hybrid mechanism. Reproduced with permission from Ref. [135], copyright 2019 American Chemical Society. (e) Polarization-controlled PVDF-based TENG-PENG hybrid generator for effectively harvesting the human footstep energy. Reproduced with permission from Ref. [165], copyright 2020 Elsevier B.V. (f) A miniaturized freestanding kinetic impact-based nonresonant TENG-EMG hybrid generator for harvesting the human-induced vibrations. Reproduced with permission from Ref. [134], copyright 2020 Elsevier B.V. (g) A self-cleaning flexible hybrid energy harvesting system integrated by an F-OSC and an autonomous single-electrode TENG. Reproduced with permission from Ref. [172], copyright 2019 Elsevier B.V. (h) A hierarchically patterned self-powered sensor for multifunctional tactile sensing. Reproduced with permission from Ref. [177], copyright 2020 American Association for the Advancement of Science.

In most situations, PENGs are extensively investigated as a common compensation of energy harvesters in the hybrid integrated systems, and the use of a piezoelectric element as a sensor has also been studied for the detection of more diversified signals [160–162]. As shown in Figure 6(a), Gong et al. proposed a hybrid generator by combining the high voltage from a nonpiezoelectric meso-poly(lactic acid) (meso-PLA) electret-based TENG (E-TENG) and the relatively high current from a double-layered poly(l-lactic acid)- (PLLA-) based PENG [109]. The triangle-waveform E-TENG exhibits the largest *V*_oc_ and *I*_sc_ at the same pressure compared with other shapes of E-TENG, e.g., trapezoidal waveform and square waveform. The output power of the hybrid generator is 0.31 mW, which is 11% higher than that of the PLLA-based PENG. Besides, the hybrid generator can also be woven into the shape of a fabric format as E-skin for wearable applications. During an elbow bending test, the PLA-based woven E-skin device can generate an output voltage of 35 V and a short-circuit current of 1 *μ*A. With the advantages of biocompatibility, ease of fabrication, and relatively high output power, the hybrid generator shows great promise for future E-skin development such as biodegradability.

As depicted in Figure 6(b), Zou et al. presented a spacer-free hybrid generator with a self-arched structure based on the effect of stress mismatch at the interface of two polymers [163]. The self-arched nanogenerator (SANG) consists of a self-arched layer and a flat layer. The Ecoflex film in the self-arched layer and the Al film in the flat layer form a TENG, and the PVDF film with Ag electrodes forms the core part of the PENG. With different mass ratios of PDMS and Ecoflex, the bending degrees of the self-arched structure of the SANG will be different. More PDMS in the self-arched structure leads to a larger bending degree. The hybrid mode of the device can help the SANG achieve a significantly higher current output of 500 nA than that of the triboelectric mode (175 nA) and the piezoelectric mode (250 nA). In addition, the SANG shows a more clear and stable hybrid signal than the individual parts, and hence, the SANG has considerable sensitivity and stability to act as a proper device for sensing pulse waveform of the radial artery.

Next, as shown in Figure 6(c), Syu et al. developed a biomimetic hybrid self-powered sensor (BHSS) [164], which produces an average *V*_oc_/*I*_sc_ of 15 V/115 nA and power density of 675 *μ*W m^–2^ under a cyclically deformed strain of 0.5% at 2 Hz. A piezoelectric PVDF nano-microfiber (NMF) is deposited on the prepatterned printed circuit board (PCB) substrate in the in situ poled fashion of aligned dipoles, forming the PENG part of the BHSS. The PDMS film with Mytilidae nanostructured patterns via the soft transfer molding technique and the Cu layer compose the two triboelectric layers of the TENG part. Therefore, the self-powered wearable sensor can be achieved through the hybridization of piezoelectric and triboelectric mechanisms. Furthermore, an intelligence glove is developed based on the BHSS and used as a loading force sensor. The synergetic collaboration of individual components in the hybrid wearable device strengthens the energy harvesting performance and enriches the sensing capability of a variety of signals.

As illustrated in Figure 6(d), Zhu et al. reported a self-powered and self-functional sock (S^2^-sock) based on a poly(3,4-ethylenedioxythiophene) polystyrene-sulfonate- (PEDOT:PSS-) coated textile-TENG integrated with a PZT-based piezoelectric sensor [135]. PEDOT:PSS is adopted as the electrode material to incorporate good conductivity and high stability into a normal fabric, which enables the TENG with a larger voltage of 196 V compared with the original fabric of 55 V. After integrating the thin PZT chips into the S^2^-sock, the TENG and PENG can work synergistically for footstep energy harvesting and self-powered sensing. In terms of walking gait monitoring and contact force analysis, the S^2^-sock is fabricated with multisectoral PEDOT:PSS patterns and embedded thin PZT chips, showing great promises in smart home, sports monitoring, healthcare, etc.

Lee et al. reported another hybrid generator by electric polarization-controlled PVDF-based TENG and PENG for effectively harvesting the vibrational energy from the human footsteps, as indicated in Figure 6(e) [165]. The proposed TENG-PENG hybrid generator consists of two PVDF films, three Al electrodes, and two acrylic supports. The upper TENG component is vertically stacked on and electrically connected to the lower PENG component, which makes the up-down polarized hybrid generator produce the largest power of 127 *μ*W due to the modulated surface potential and negative piezoelectricity of PVDF. When three hybrid generators were embedded at the forefoot, arch, and heel positions in a shoe insole, the whole insole device can generate enough energy to light up LEDs and drive a wireless pressure sensor during normal walking which can be further applied for diagnostic healthcare.

In addition to the integration with PENGs, TENGs can also be combined with other mechanisms for the wearable application [166, 167]. Among these mechanisms, EMGs normally exhibit high current outputs that would be a good complement to the TENG's high voltage outputs [108, 168, 169]. Hybridizing multiple mechanisms such as TENG and EMG into an energy harvester is a suitable approach for improving the power conversion efficiency of human-induced mechanical excitations. As shown in Figure 6(f), Rahman et al. reported a highly miniaturized freestanding kinetic impact-based hybrid generator as an effective energy harvester for various human-induced vibrations [134]. The rational integration of EMG and TENG into a common mechanical system can improve the power generation capability of the hybrid generator under the same mechanical input. For the testing using a shaker at 5 Hz, the EMG and the TENG can produce a maximum power of 102.12 mW and 171.13 *μ*W, respectively. For different body-worn positions of the hybrid generator under walking and slow running activities, a storage capacitor can be effectively charged up to various voltage levels according to the motion-induced accelerations. Moreover, two digital temperature-humidity meters and an array of commercial LEDs are simultaneously powered by the random vibrations of human motions. With the aid of a customized power management circuit, the output can be used to power modern electronics like smartphones, smartwatches, and wireless temperature sensors.

In terms of miniaturized energy harvesting systems, integrating TENG with organic SC becomes a significant approach to collect the solar energy owing to its flexibility that can be seamlessly integrated with human and the compatibility with large-scale and low-cost manufacturing techniques [124, 170]. The integration of TENGs and flexible SCs enables the simultaneous collection of human mechanical and solar energy [171]. As illustrated in Figure 6(g), Ren et al. developed a novel self-cleaning flexible hybrid energy harvesting system which includes a groove-shape micro-/nanostructured haze thin film (GHF), a flexible power management circuit, and a hybrid generator composed of a flexible organic SC (F-OSC) and an autonomous single-electrode TENG (AS-TENG) via one common electrode [172]. This hybrid system would collect both solar and mechanical energies through the top F-OSC and the bottom AS-TENG that can simultaneously utilize the large current of the SC and the high voltage of the TENG by the flexible power management circuit. In addition, GHF with optical property, large surface area, and superhydrophobicity has been introduced into the hybrid generator, serving not only as a triboelectric layer to increase the surface charge density of the TENG but also as a light-trapping layer to improve the photoelectric conversion efficiency of the SC. The hybrid generator can be integrated into the garment in an embedded manner as a high-performance wearable hybrid energy harvesting system. The above results provide a solution to break environmental constraints, thereby maximizing the collection and conversion efficiency of ambient energy and improving the stability of hybrid generators in practical applications.

Other than using hybrid generators for effective energy harvesting, they can also be adopted as fascinating sensors to simultaneously detect various stimuli such as pressure and thermal variations in a single device using ferroelectric or organic thermoelectric materials [173, 174]. Meanwhile, they can be further employed as important components of E-skin, which yields a popular research direction to develop wearable sensors with multifunctional sensing capability [175, 176]. Integrating TENG with other mechanisms could provide a feasible solution to realize skin-like sensing systems. Wang et al. presented a multifunctional, tactile, and self-powered sensor that enables pressure, temperature, and material sensing, as shown in Figure 6(h) [177]. The sensor exhibits the form of a multilayer stack: a hydrophobic PTFE film as the triboelectrification layer, two Cu sheets coated with the Ag nanowire film as electrodes, and a sponge-like graphene/PDMS composite as the responsive component for pressure and temperature sensing based on the piezoresistive and thermoelectric effects. The sensing for pressure is achieved by measuring the contact resistance between the electrode and the conducting graphene/PDMS sponge. According to the thermoelectricity, the device shows the temperature sensing properties when contacting with an object of different temperature. The sensor exhibits pressure sensitivity and self-powered temperature sensing accuracy of 15.22 kPa^−1^ and 1 K, respectively. At the same time, to realize material identification, the triboelectrification phenomenon is used to show the natural physical property of materials, by leveraging the lookup table algorithm. The proposed device can infer 10 different flat materials, which opens a new path for using self-powered sensors in tactile sensing and material identification. As a result, this hierarchically patterned self-powered sensor offers a promising approach for multifunctional sensing with potential applications in wearable electronics and robotics.

### 6.2. Implantable Hybrid Generators

Implantable devices experienced tremendous growth which are becoming indispensable medical tools for improving the quality of thousands' life, which can contribute significantly to helping us cognize the diseases of organs and support real-time treatment [15, 50, 178, 179]. Since the motion energy of organs could be detected and harvested by nanogenerators, their outputs are strongly related to many biomedical signals, such as electrocardiogram (ECG) [180], heart rate [181], blood pressure [182], and respiratory motion [183]. Therefore, nanogenerators show great potential in the implantable electrical stimulation systems, such as the brain, muscle, and peripheral nervous system [43, 184–187]. Due to the above-mentioned superior merits, TENGs with appropriate material and structural engineering have been extensively investigated for diversified implantable applications [4, 188, 189]. By harvesting the mechanical deformation energy from the contraction of the heart, brain, and vagus nerve, stimulation has been successfully realized by PENGs and TENGs, respectively [190, 191]. Similar to the evolution trend of the wearable sensor system, the integration of neural interfaces, sensors, and other functional devices is emerging to form a self-powered implantable system with a variety of applications.

**Figure 7:**
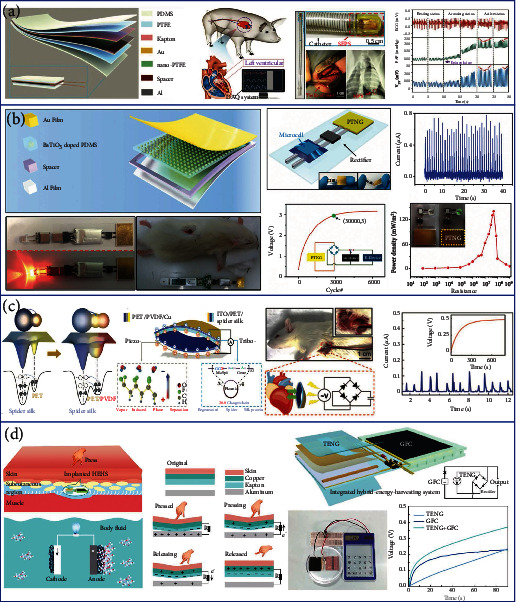
TENG-based hybrid generators for implantable devices. (a) Transcatheter self-powered ultrasensitive endocardial pressure sensor. Reproduced with permission from Ref. [192], copyright 2020 Wiley-VCH. (b) A packaged self-powered system with universal connectors based on a triboelectric-piezoelectric hybrid generator. Reproduced with permission from Ref. [193], copyright 2015 Wiley-VCH. (c) A “self-matched” triboelectric-piezoelectric generator using vapor-induced phase-separated poly(vinylidene fluoride) and recombinant spider silk. Reproduced with permission from Ref. [194], copyright 2020 Wiley-VCH. (d) A hybrid energy harvesting system based on a TENG and a glucose fuel cell for potential in vivo applications. Reproduced with permission from Ref. [195], copyright 2020 Springer Nature.

On the basis of TENG, Liu et al. reported a miniaturized, flexible, ultrasensitive, and self-powered endocardial pressure sensor (SEPS) based on TENG for real-time endocardial pressure (EP) monitoring in Figure 7(a) [192]. SEPS can convert the energy of blood flow within the heart chambers into electricity. The electric outputs of the device can indicate the physiological and pathological cardiovascular status, including EP, ventricular fibrillation, and ventricular premature contraction. In order to imitatively evaluate the function of the SEPS for real-time biomedical monitoring, a male adult Yorkshire pig (40 kg) as the animal model is implanted with the SEPS for detecting the signals of ECG and the femoral arterial pressure (FAP). When the FAP reaches and maintains at its upper limit (active status), the peaks of SEPS outputs present coinstantaneous tiny fluctuations along with the systolic FAP (arrows), implying an excellent sensitivity of the device in response to the subtle changes of endocardial pressure. Therefore, the device promotes the development of miniature implantable medical sensors for monitoring and diagnosis of cardiovascular diseases. With the robust self-powered capability, the SEPS exempts the necessity of onboard batteries, showing great potential in monitoring and diagnosing cardiovascular diseases.

A few challenges such as the complicated implanted environment, limited space, large internal impedance, conversion efficiency, and material biocompatibility/biodegradability, still remain to be overcome by the current implantable TENGs before they can be widely put into practical applications. Specifically, the output of the implantable TENGs needs to be enhanced for effectively driving implantable medical devices. Therefore, combined with the versatile transducing mechanisms, hybrid generators can promote the development of self-powered implantable medical devices.

Recently, the integration of TENGs and PENGs offers an efficient approach to enhance the energy conversion efficiency. As shown in Figure 7(b), Shi et al. presented a piezoelectric and triboelectric hybrid nanogenerator (PTNG) to realize a packaged self-powered system (PSNGS) [193]. The triboelectric part is working based on the vertical contact-separation mode, whereas the contact layers are the BaTiO_3_-doped PDMS film and the Al film. Besides, the polarized BaTiO_3_-doped PDMS film can generate piezoelectric potential under stretched status, which can be used to enhance the triboelectric output through a synergistic effect. The PSNGS mainly consists of four parts: the PTNG as a power source, a rectifier, a microbattery, and a flexible substrate. When further considering the implantable or portable applications, the size of the PSNGS should be strictly controlled. Under the periodic motion of the linear motor, the voltage of the microbattery can be charged by the PTNG from 0.5 to 3 V within 30,000 cycles. After applying the PSNGS as the sustainable power source, an electronic thermometer can be driven and placed into the incision site to detect the subcutaneous temperature of a live rat.

Huang et al. reported a “self-matched” TENG-PENG hybrid nanogenerator using vapor-induced phase-separated poly(vinylidene fluoride) and recombinant spider silk to harvest the mechanical energy more efficiently, as illustrated in Figure 7(c) [194]. The electrons occupying the specific protein molecular orbits tend to transfer to the empty orbits of PET when they contact. Furthermore, the number of transferred electrons is proportional to the difference of potential well depths between the two materials (surface potential difference). PVDF as a flexible piezoelectric and biocompatible polymer has the capability to change the surface electron potential of the coupling material under mechanical deformation. Thus, the enhanced difference in potential well depths between the spider silk and the PET/PVDF can significantly increase the number of transferred electrons and thereby boost the energy output. After encapsulating the device in a silk-based package, it can be implanted in the chest of a Sprague-Dawley rat for heartbeat energy harvesting and monitoring. The fluctuating peak current from the heartbeats can power a 4.7 *μ*F capacitor to about 0.5 V within 5 min, which indicates that the “self-matched” TPNG could be implanted in various human tissues such as the stomach, chest, and bladder for self-powered medical monitoring and treatment.

As shown in Figure 7(d), Li et al. reported a hybrid energy harvesting system (HEHS) consisting of a TENG and a glucose fuel cell (GFC) on a flexible PET substrate for simultaneously harvesting the biomechanical energy and the biochemical energy in simulated body fluid [195]. The body fluid containing glucose molecules penetrates into the active materials and then participates in the redox reaction around the anode electrode of the GFC. The lost electrons of glucose migrate from anode to cathode and are captured by dissolved oxygen in the body fluid. This process converts the biochemical energy in glucose into electric energy. The TENG and the GFC are connected in parallel to enhance the combined output current because of the higher voltage but similar current of the TENG (22 V) compared to the GFC. For charging capacitors, the HEHS has an obviously faster charging rate than the individual TENG and GFC, achieving a higher voltage (0.37 V) within 90 s. The collected power can drive a calculator or light up a green LED pattern. As a result, this study provides a feasible method to harvest energy from multiple sources, showing great potential as an implantable power source to drive low-power electronic devices. In short summary, implantable hybrid generators exhibit advantages for powering implantable medical electronics with excellent output performance, high power density, and good durability.

## 7. Power Management and Energy Storage

Accounted for the high-voltage-low-current and large internal impedance characteristics of conventional TENGs, different power management strategies have been developed for TENGs to productively improve the energy conversion and energy storage efficiency [196]. For the hybrid generators, specific consideration in a power management circuit (PMC) should be taken into account due to the more complicated configuration and output characteristics, such as the impedance match of different generator components, the mixing AC/DC outputs from different components, and the impedance match of the hybrid generators and storage units. Therefore, achieving high-efficiency power management and energy storage is essential to realize high-performance and self-sustainable systems.

### 7.1. Power Management

**Figure 8:**
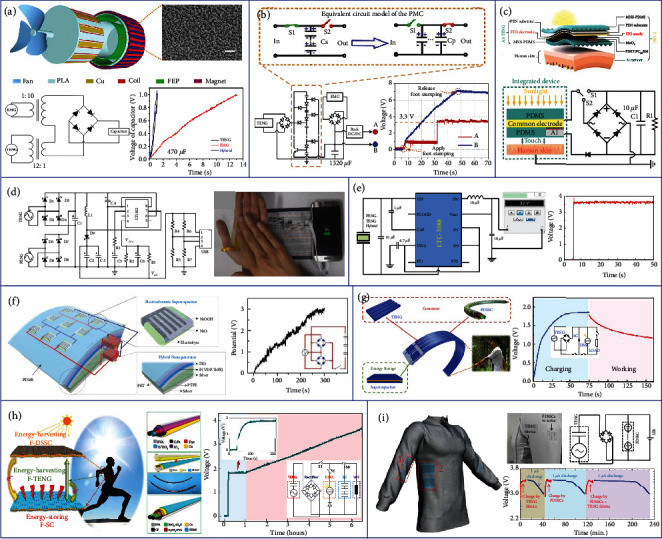
Power management and energy storage for TENG-based hybrid generators. (a) A PMC with transformer-based impedance matching for a TENG-EMG hybrid generator. Reproduced with permission from Ref. [200], copyright 2017 American Chemical Society. (b) A transistor-controlled PMC for automatic parallel-series capacitor transformation for better impedance match of different components. Reproduced with permission from Ref. [201], copyright 2019 Elsevier B.V. (c) A typical PMC on a flexible substrate for a TENG-SC hybrid generator. Reproduced with permission from Ref. [172], copyright 2019 Elsevier B.V. (d) A customized PMC with a stable USB output port based on a TENG-PENG hybrid generator. Reproduced with permission from Ref. [202], copyright 2019 Elsevier B.V. (e) A PMC using bridge rectifiers and adjustable buck chopper for regulating the pulse outputs into stable DC outputs. Reproduced with permission from Ref. [107], copyright 2018 Elsevier B.V. (f) A self-charging power package by the integration of a TENG-PENG hybrid generator and a micro-supercapacitor array. Reproduced with permission from Ref. [207], copyright 2019 Wiley-VCH. (g) A self-charging power bracelet integrating a flexible TENG with multiple fiber-shaped dye-sensitized SCs and supercapacitors. Reproduced with permission from Ref. [208], copyright 2019 Elsevier B.V. (h) A self-charging power textile by the integration of a fiber-shaped TENG-SC hybrid generator and multiple energy-storing fiber-shaped supercapacitors. Reproduced with permission from Ref. [209], copyright 2016 American Association for the Advancement of Science. (i) A self-charging power textile composed of a fabric TENG, fiber-shaped dye-sensitized SCs, and a LIB. Reproduced with permission from Ref. [210], copyright 2016 Wiley-VCH.

One of the most common TENG-based hybrid generators is the TENG and EMG hybrid, in which the power management strategy is highly important due to the significant impedance mismatch of TENG and EMG [197–199]. As shown in Figure 8(a), Cao et al. developed a PMC with an impedance matching strategy for a TENG-EMG hybrid generator [200]. The PMC consists of two commercial transformers and a bridge rectifier. One transformer with the ratio of 1 : 10 is connected to the EMG, and the other transformer with the ratio of 12 : 1 is connected to the TENG, in order to achieve an impedance match between the two components. After that, the two outputs are connected in series into the bridge rectifier for capacitor charging. It is demonstrated that only 0.8 s is required for the hybrid generator to charge up a 470 *μ*F capacitor to 1 V, showing great potential to realize self-sustainable systems using hybrid generators with efficient PMCs.

Another common strategy for power management of TENG-EMG hybrid generators is using parallel-series switchable capacitors. As illustrated in Figure 8(b), Chen et al. proposed a transistor-controlled PMC for automatic parallel-series capacitor transformation to overcome the huge impedance mismatch between TENG, EMG, and the energy storage unit [201]. In the PMC, automatic switchability is designed through combining a transistor with transition capacitors (small capacitance) and diodes for the rectified triboelectric output. The transistor functions as an electronic switch to automatically change the connection scheme of the capacitors from series connection in charging states to parallel connection in discharging states. In the discharging state, all the small capacitors are connected in parallel to charge an energy storage capacitor with large capacitance. Thus, the output voltage of the TENG can be lowered by *N* times, and the output charge can be improved by *N* times (*N* is the number of small capacitors used in the PMC). In this way, the impedance match between the TENG and the energy storage capacitor can be greatly improved. As for the EMG, it is directly connected to the energy storage capacitor through a rectifier due to a better impedance match. In addition, a DC/DC buck converter unit is further connected to the energy storage capacitor to achieve a stable output voltage of 3.3 V. After applying foot stamping to the TENG-EMG hybrid generator at ~2 Hz, a capacitor of 1.32 mF can be charged up to 7 V within 40 s.

While TENG, EMG, PENG, and PyENG normally show AC output characteristics, TEG and SC exhibit DC characteristics that do not require rectifiers for output regulation. As depicted in Figure 8(c), Ren et al. presented a PMC on a flexible substrate for a hybrid generator composed of an autonomous single-electrode TENG and a flexible organic SC [172]. A bridge rectifier is adopted to convert the AC outputs from the TENG into DC outputs before charging a capacitor, while the DC outputs from the SC can be directly connected. In the PMC, a diode is used to prevent the TENG currents to go through the SC. The PMC demonstrated here is the basic power management strategy when there are mixing AC and DC outputs in a hybrid generator, yet it is worth noting that the charging efficiency could be further enhanced by adopting the impedance matching strategy for the TENG as discussed above.

In the practical application of powering small electronics, continuous and stable DC output is always required, which exerts higher demand for traditional power management. As indicated in Figure 8(d), Rasel et al. developed a customized PMC with a universal serial bus (USB) port as the stable DC output for a TENG-PENG hybrid generator [202]. The PMC consists of two respective bridge rectifiers for the TENG and the PENG, an energy storage capacitor, a DC/DC converter, and a USB port for stable output. To achieve quick response time and minimize the power dissipation in the PMC, a high-conversion-efficiency DC/DC voltage converter is employed (LT1302, Linear Technology). Through the PMC, the time-varying AC outputs from the hybrid generator are converted into constant DC voltage, which can be then used to charge a commercial pedometer, trimmer, pocket Wi-Fi router, and smartphone directly. Similarly, another stable DC power source by combining a TENG-PENG hybrid generator with a customized PMC is presented in Figure 8(e) by Zhao et al. [107]. Two low-loss bridge rectifiers are integrated with an adjustable buck chopper, forming the PMC for regulating the pulse signals from the hybrid generator into stable DC outputs. After connecting the hybrid generator with the PMC, a stable DC power source with a 3.6 V output can be achieved to drive different commercial electronics such as calculator, multifunctional meter, and RF-wireless temperature sensor.

### 7.2. Energy Storage

To efficiently store the converted electrical energy from hybrid generators, various energy storage units such as supercapacitors and batteries have been developed. Specifically, flexible energy storage units have received rapid advancement for wearable applications in recent years [203–206]. To construct functional and self-sustainable systems, energy storage units that are developed under the same platform with the hybrid generators are more favorable and are attracting increasing research attention. As illustrated in Figure 8(f), Qin et al. demonstrated a self-charging power package through the integration of a TENG-PENG hybrid generator and an electrochromic microsupercapacitor array [207]. Benefitting from the electrochromic property, the power package is able to indicate its charging status with color changes, which offers more convenience in monitoring the charging process. The electrochromic microsupercapacitors with the Ag nanowires/NiO as the electrode materials exhibit a high capacitance of 3.47 mF cm^−2^ and a good cycling performance. By the high output performance of the hybrid generator (150 V and 20 *μ*A) under human palm impact, the microsupercapacitor array can be self-charged to 3 V within 300 s for lighting up LED and driving small electronics.

Later, Song et al. developed an elastic self-charging power bracelet, as shown in Figure 8(g), which integrates a flexible TENG and several fiber-shaped dye-sensitized SCs with supercapacitors, in order to simultaneously harvest the mechanical and solar energy [208]. The supercapacitors as the energy storage units are fabricated under the same platform to store the energy from the TENG-SC hybrid generator. Due to the good flexibility, the self-charging power bracelet can be conveniently tuned and connected with other electronics as a sustainable power source. For charging three series-connected supercapacitors, the voltage on the supercapacitors can increase smoothly to ~1.8 V in 43 s, which can steadily drive the operation of an electronic watch for 85 s.

With the extensive innovation in wearable electronics in the past few years, textile-based generators and energy storage units have also received rapid development. As depicted in Figure 8(h), Wen et al. demonstrated a self-charging power textile through the integration of a fiber-shaped TENG-SC hybrid generator and multiple fiber-shaped supercapacitors, for simultaneous biomechanical/solar energy collection and storage [209]. When the supercapacitor is only charged by the SC, its voltage can be increased to 1.8 V in about 69 s, which remains constant afterward due to the low output voltage of the SC. The integrated TENG can compensate for this weakness and further charge up the supercapacitor to a higher voltage level. Since both the hybrid generator and the supercapacitor are developed under the same fiber platform, the self-charging power textile offers great convenience in the systemic integration and can be easily woven into normal clothes to enable the application in smart/power wearables.

In Figure 8(i), Pu et al. developed another power textile by integrating a fabric TENG (grating structure) and fiber-shaped dye-sensitized SCs (FDSSCs) with a lithium-ion battery (LIB) as the energy storage unit [210]. The TENG is designed with the grating structure and fabricated by Ni plating, so as to convert the common low-frequency human motions into high-frequency electrical outputs. Then, the outputs from the TENG are first connected to a bridge rectifier and then in parallel with seven FDSSCs (with an output voltage of ~5 V in series) to charge the LIB. To compare the charging performance, the LIB is charged by the fabric TENG, the FDSSCs, and the hybrid generator for 10 min, respectively, followed by a constant discharge at 1 *μ*A. The results demonstrate that the discharge curves can last for 28, 59, and 98 min, respectively, showing the synergetic and improved charging effect of the hybrid generator. It can be observed that in the above energy storage examples, most of the TENGs are just simply connected to energy storage units after bridge rectifiers, which induces a great impedance mismatch between the TENG component and the energy storage unit as well as other energy harvesting components. Therefore, in a realistic application, efficient power management strategies such as impedance matching should be performed to further improve the charging performance and achieve maximum conversion efficiency.

## 8. Functional and Self-Sustainable Hybridized Systems

With the proper power management and energy storage discussed in the above section, functional and self-sustainable hybridized systems without external power supplies can be eventually realized by further combining the hybrid generator-based power packages with functional components [211–216]. Other than just serving as the power sources, the energy harvesting components in hybrid generators can also function as self-powered active sensors to reduce the overall power consumption of the hybridized system.

**Figure 9:**
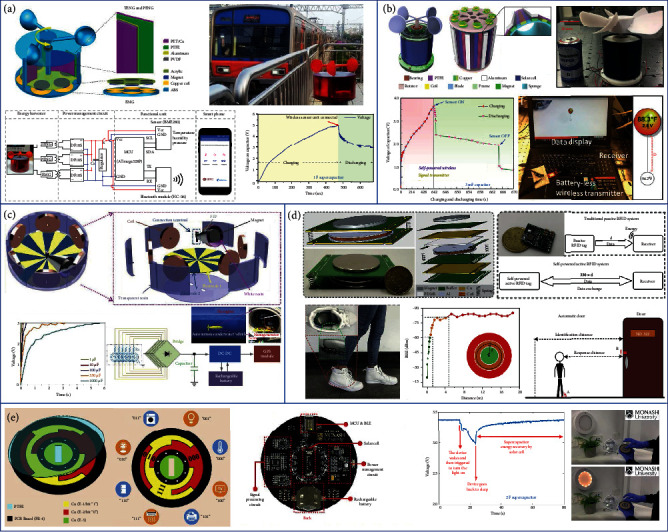
Functional and self-sustainable hybridized systems. (a) A wireless self-powered environmental monitoring system by harvesting wind energy from the surroundings. Reproduced with permission from Ref. [217], copyright 2018 Elsevier B.V. (b) A self-powered disaster monitoring system that can simultaneously harvest the ambient wind and solar energy. Reproduced with permission from Ref. [218], copyright 2018 Elsevier B.V. (c) A self-powered tracking system for monitoring marine equipment's position and attitude by blue energy harvesting. Reproduced with permission from Ref. [219], copyright 2020 Elsevier B.V. (d) A self-powered RFID tag by integrating the RFID with a TENG-EMG hybrid generator. Reproduced with permission from Ref. [201], copyright 2019 Elsevier B.V. (e) A self-sustainable control system for smart home interactions with different appliances. Reproduced with permission from Ref. [220], copyright 2020 Elsevier B.V.

As shown in Figure 9(a), Rahman et al. demonstrated a wireless self-powered environmental monitoring system by harvesting the wind energy from the surroundings [217]. The self-powered system is developed from a windmill-shaped hybrid generator that comprises a TENG, a PENG, and an EMG. All the three generators are integrated into a common 3D printed cylinder structure to convert the wind-induced rotation energy into electricity. It is shown that when the wind speed is 6 m s^−1^, the blade-based TENG and PENG can produce a maximum output power of 1.67 mW (at 10 M*Ω*) and 1.38 mW (at 330 k*Ω*), respectively. In the meanwhile, the multiple magnet-based EMG can generate a maximum output power of 268.6 mW at an optimal load resistance of 180 *Ω*. The hybrid generator is further combined with a customized PMC (composed of transformers, rectifiers, a supercapacitor, a voltage regulator, and a switch), a microcontroller unit, an environmental sensor, and a Bluetooth module for constructing the wireless self-powered system. A 1 F supercapacitor can be charged up to 5 V by the hybrid generator in 465 s, which can then drive the whole system for about 195 s. The wirelessly transmitted data from the system can be received by a smartphone, showing the real-time monitoring information of the ambient parameters.

To enable self-powered disaster monitoring, Qian and Jing developed a self-powered system that can harvest the ambient mechanical wind and solar energy simultaneously, as indicated in Figure 9(b) [218]. The wind energy harvester has a rotating structure that integrates sliding TENGs and EMGs. Next, a waterproof and flexible SC is integrated into the outer frame of the structure for solar energy harvesting. The developed self-powered disaster monitoring network includes three stages: energy harvesting-enabled self-powered monitoring, information collection and data transmission, and data receiving and response. To demonstrate the practical application, a self-powered temperature monitoring system is developed by combining the hybrid generator with a temperature sensor, a microcontroller, an RF transceiver, and an antenna. Under a rotation rate of 2100 rpm, a 3 mF capacitor can be charged to 3.64 V in 642 s, which can continuously drive the system for ~22.6 s. Then, a receiver equipped with a computer can collect and display the temperature and the distance (*D*) information for real-time monitoring.

Due to the complicated and offshore environment, self-powered systems are of great importance in ocean-relative applications. As illustrated in Figure 9(c), Gao et al. presented a self-powered tracking system for monitoring marine equipment's position and attitude, by combining an EMG-powered global position system (GPS) module with a TENG-based inertial sensor [219]. The TENG and EMG are integrated into a rotating gyro structure, which shows good performance in energy harvesting and multiple-direction sensing. Under excitation, a 1000 *μ*F capacitor can be charged to 3 V by the EMG in around 6 s. To provide a stable DC voltage to the GPS module, a PMC with the functions of AC-DC conversion, DC-DC voltage regulation, energy storage, and release control is built. With the constant power supply, the GPS module can communicate with satellites for real-time position tracking. Furthermore, the self-powered system is packaged inside an autonomous underwater vehicle (AUV) and validated in the Huanghai Sea under actual working scenarios, showing great potentials of hybrid generators in blue energy applications.

To solve the limited working distance of the traditional radio frequency identification (RFID) tags while still maintaining battery-less characteristics, Chen et al. proposed a self-powered RFID tag by the integration with a TENG-EMG hybrid generator, as depicted in Figure 9(d) [201]. The hybrid generator can effectively harvest the human biomechanical energy with a maximum power density of 6.79 W m^−2^. In addition, a PMC with an impedance matching strategy by the parallel-series transformation of capacitors is developed, which can improve the capacitor charging efficiency by approximately 50%. With the harvested energy from human working, the hybrid generator-driven self-powered RFID tag shows a significantly enhanced working distance. Furthermore, based on the received signal strength indicator (RSSI) on the receiver side, the distance from the tag to the receiver can also be calculated and used for automatic door control in smart home relative applications.

In the rapidly expanded IoT era, developing self-sustainable human-machine interaction systems is of great significance for information exchange and communication. As illustrated in Figure 9(e), Qiu et al. demonstrated a self-sustainable control system for smart home interactions [220]. The control system consists of a photovoltaic cell as the power source, a sliding TENG as the control interface, and a PMC for power management, signal processing, and wireless communication. In the TENG control interface, a 3-bit binary-reflected Gray code (BRGC) pattern is designed with two sensing electrodes, where one of them represents the bit “0” and the other one represents the bit “1.” A third electrode located in the middle of the control interface is adopted to differentiate the inward and outward sliding directions. When the finger slides across different patterns, the unique output signals can be detected for different appliances' control. After activation, the overall energy consumption of the control system for sensing and wireless communication is about 18 mA at 3.3 V. Then, the consumed energy of each control operation can be restored in 55 s by the photovoltaic cell, showing great potential for self-powered control and interactions.

## 9. Conclusions and Prospects

**Figure 10:**
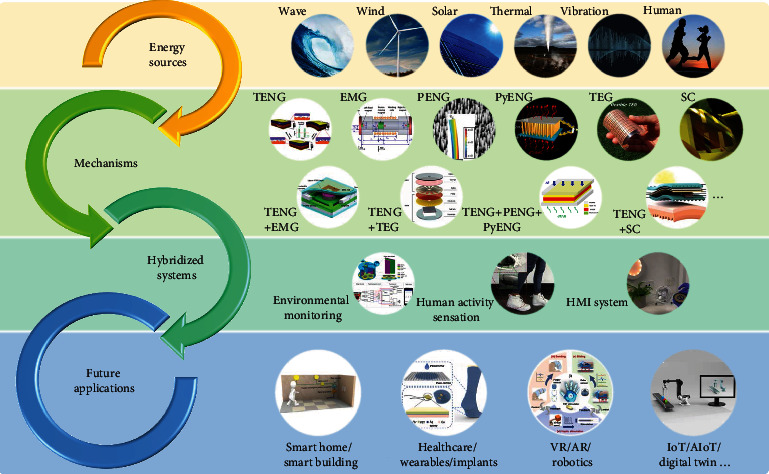
Prospects on the development and applications of hybridized systems. Reproduced with permission from Refs. [11, 58, 64, 133, 135, 142, 172, 201, 217, 220–225, 228, 229], copyright 2012 Elsevier B.V., 2015 IEEE, 2006 American Association for the Advancement of Science, 2012 American Chemical Society, 2016 American Chemical Society, 2014 Royal Society of Chemistry, 2017 Springer Nature, 2018 Wiley-VCH, 2018 Elsevier B.V., 2019 Elsevier B.V., 2018 Elsevier B.V., 2019 Elsevier B.V., 2020 Elsevier B.V., 2020 Springer Nature, 2019 American Chemical Society, 2020 American Association for the Advancement of Science, and 2020 Springer Nature.

In the rapidly developing IoT era, TENG-based hybrid generators (e.g., TENG integration with one or more of the generators such as EMG, PENG, PyENG, TEG, and SC) provide a promising solution to enable the realization of functional and self-sustainable systems, by synergistically collecting the single-source and multisource energies. In this review, we systematically summarize the recent development of TENG-based hybrid generators and hybridized systems in terms of outdoor, indoor, wearable, and implantable applications. As illustrated in Figure 10, various forms of energies in the environment (such as mechanical, thermal, and solar) can be effectively converted into electrical energy through employing the appropriate transducing mechanisms and their synergistically integrated hybrids [221–225]. For the single-source mechanical energy harvesting, TENG can be integrated with EMG and PENG under the same actuation scheme for compensating for the shortcomings of each generator and improving the energy conversion efficiency. As for the multisource energy harvesting, TENG-integrated PyENG, TEG, and SC under the same platform will be more favorable in terms of facile systemic integration and structural complexity reduction. By further integrating the hybrid generators with efficient PMCs, energy storage units, and other functional components, self-sustainable hybridized systems can be realized for a wide range of applications, e.g., environmental monitoring, human activity sensation, human-machine interaction, smart home, healthcare, wearables, implants, virtual/augmented reality (VR/AR), robotics, IoT, and artificial intelligence of things (AIoT) [142, 226–229].

Though the future is promising, there are still some challenges remaining to be further addressed in the current developed TENG-based hybrid generators and hybridized systems. First, further power enhancement of the hybrid generators is still inevitably desired, through maximizing the output performance of each generator and synergic integration. In most of the demonstrated self-powered systems, the whole system can only function for a short period of time but requires a much longer charging period. This intermittent operation mode highly limits their applicability in some crucial applications such as toxic matter monitoring, where intensive and continuous operations are required. Second, the long-term durability and the performance robustness under different ambient conditions are major concerns for TENG-based systems. The involved periodical contact/friction of triboelectric materials and the high susceptibility to the ambient humidity of TENGs raise grand challenges for the material and structural design. Third, due to the large impedance mismatch between different energy harvesting components and energy storage units, effective power management is of great importance in achieving higher conversion efficiency. Moreover, TENG enhancement through optimized material/structural/circuitry design can be implemented before the power management for the hybrid generator to obtain a higher output performance. Last but not the least, for the long-term functionality and stability of the hybridized systems, energy storage units such as supercapacitors and batteries should possess high energy storage density and reliability. Besides, energy storage units that are developed under the same platform as the hybrid generators (e.g., flexible, stretchable, and textile) will be more favorable for the convenient integration of the whole hybridized system. In this regard, the bright future of the IoT era will be greatly beneficial by the realization of the functional and self-sustainable hybridized systems in diverse application areas.
